# Rabies in Cattle1This report comes to us as prepared by Mr. Eddy, of the senior class of the Veterinary Department of the Ohio State University, as a part of the system of instruction adopted.

**Published:** 1899-12

**Authors:** William Eddy

**Affiliations:** Senior Student in Veterinary Department of Ohio State University, Columbus, Ohio


### REPORTS OF CASES.

### RABIES IN CATTLE.[1]

By William Eddy,
SENIOR STUDENT IN VETERINARY DEPARTMENT OF OHIO STATE UNIVERSITY, COLUMBUS, OHIO.

On October 3d we were called to a dairy-farm, about three miles from the college, to see a two-year old Jersey heifer suffering from what the owner thought to be indigestion from eating sugar-cane. We found the animal down, paralyzed behind, but able to drag herself around with forelimbs; had eaten nothing for four days prior to our call; temperature, 105.5°; pulse, 86; respiration, 16; peristalsis good; flow of milk had dried up entirely. Owner stated that she had been very irritable for several days past, and would attack chickens or the family dog whenever they came into the yard where she was confined. We suspected rabies, and questioned owner very

[1] This report comes to us as prepared by Mr. Eddy, of the senior class of the Veterinary Department of the Ohio State University, as a part of the system of instruction adopted.

closely as to whether he knew of her having been bitten at any time or not by a dog. By close questioning we found that he had lost a dog about five weeks previous from what he supposed to be distemper. He only noticed her being sick a few days, but recollected seeing her snap at the cattle when they came up to the trough, which was close to the house, but thought nothing of this, as she had always been cross toward the cattle. While sick she had shown no disposition to attack persons, but would wander off from home, be gone for several hours, and when she returned she showed great exhaustion. The afternoon she died he used her for several hours to hunt rats in the hay barn, and she seemed perfectly natural, except that she was very weak and much emaciated. He found her dead under the barn next day.

We requested owner to notify us when the heifer died, as we were anxious to hold a post-mortem. On the 5th we received word that the animal had died during the night. We held the post-mortem twenty hours after death; no careful notes were taken, as decomposition was beginning. Digestive and respiratory tracts seemed normal; blood was thick, tarry, and coagulated; brain much congested. We removed brain and inoculated two rabbits with an emulsion of medulla in distilled water. The inoculation was made directly into the cranial cavity.

On the following day, October 6th, we were requested to make another call, the owner stating that this animal was showing exactly the same symptoms as the other. Our patient was a two-year old Jersey heifer about the size and appearance of the first animal. The pulse was 55; respiration, 15; temperature, 101.5°; peristalsis very good, sclerotic coat of eye much congested, expression wild and staring. She showed no disposition to attack us when making an examination, but when we turned her into the barnyard she rushed furiously at the chickens. When we attempted to restrain her she would fall to the ground, throw her head to one side, refusing to rise until she caught sight of the family dog, when she would spring up and rush furiously at him, driving him from the yard. The voice was altered, being a peculiar wailing bellow. There was intense salivation, the froth streaming from both corners of the mouth. On putting her into a shed, where she was away from all excitement, she showed no very unusual symptoms, other than an occasional pawing and bellowing. She would nibble at corn fodder and manage to eat a little; water was offered her and she drank some, pushing her nose clear to the bottom of the pail. Pounds 1½ Epsom salts and ʒij gamboge were given. On the 7th she was much the same, symptoms were more pronounced, if anything, the physic had operated, respiration and temperature same as day before, but pulse had risen to 74. We were positive of rabies at the time and warned owner. We secured his permission to remove the heifer to the hospital, which was done on the 8th. While attempting to load her into a wagon she broke away and made a vicious attack on one of the team, attempting to bite the horse on the flank, but was driven off before she could inflict any wound. At the hospital we confined her in a large box-stall and observed her closely. She was easily aroused or excited by the sight or sound of dogs or chickens, but at no other time showed any furious symptoms, and at no other time did she offer to attack any person. We could go into the stall at any time and handle her without exciting her, and anyone coming into the hospital and looking at her would have thought that she was a trifle uneasy or in heat, and would never have suspected rabies. She ate very little, but showed an abnormal appetite, eating her own dung, digging up the clay floor and eating it. Her voice became more and more unnatural, being at the last only a howling wail. She became very weak and much emaciated, and died on the morning of the 11th.

A careful post-mortem, ten hours after death, was made, notes of which follow:

Condition, much emaciated, some post-mortem bloat; natural openings, slight discharge of urine from vulva, mucous membrane of which was congested, other openings normal; subcutaneous tissue, hyperæmic; musculature, dull, cloudy, and dark; abdominal cavity contained no fluid, position of viscera normal; peritoneum, clouded, smooth; paunch, congestions under parts of serous coats, mucous membrane intact, contents normal, apparently corn-fodder and pumpkins; honeycomb and muniplier were normal; fourth stomach, mucous membrane swollen, well-marked venous congestion, with few excoriations on surface, contents consisted of clay and feces; bowels, normal; liver, brittle, borders sharp, capsule a little dull and clouded, large veins congested, parenchyma lighter colored than normal; gall-bladder contained half-pint of dark, partially coagulated bile; spleen, congested; uterus contained eleven-weeks' old fœtus; thoracic cavity contained no fluid; pleura, smooth, cloudy, and dull; lungs, congested; pericardium, slightly cloudy; heart, apex sharp, coronal vessels congested, adipose tissue about heart of a semi-gelatinous consistency, frequent ecchymoses under epicardium, right side of heart filled with dark, tough, and tarry coagulated blood; myocardium, cloudy; endocardium, a little cloudy; head, sclerotic coat of eye congested; brain, congested.

Two rabbits were inoculated under dura mater, one rabbit and a dog were inoculated hypodermatically with an emulsion of the medulla in distilled water.

On October 10th we were again called to the same farm to see another case. The third animal was a very large, fine Jersey cow, five years old, showing much the same symptoms, but in a more marked degree. As she was quite vicious we did not attempt to examine her, but ordered her destroyed. We were not present when she was killed, and consequently were unable to make a post-mortem.

On October 14th another cow began showing much the same symptoms and was shot at once by the owner, who had become quite alarmed at these continued outbreaks. We did not see this animal, but from the symptoms described by the owner there is no doubt but that she was affected with rabies.

On October 22d, seventeen days after inoculation, the first rabbit died. This rabbit had been inoculated with brain substance from the first case. The little animal seemed dumpish for a few days, refusing to eat and indifferent to surroundings. It gradually became weaker, became paralyzed behind, dragging himself around with forelimbs, and on the morning of the 22d was found dead. One of the rabbits inoculated on the 11th from the second case died on the 23d, twelve days after inoculation. The symptoms were much the same as in the first rabbit, but did not last so long. This can perhaps be accounted for by the fact that this animal was rather weak, having been injured a few days after inoculation.

On October 26th, twenty-one days after inoculation from the first case, another rabbit died, having been sick five days, and showing almost exactly the same symptoms as the first. The fourth rabbit, inoculated from the second case, died October 31st, twenty days after inoculation.

Post-mortems were held on all these rabbits, and beyond emaciation and slight congestion of the brain in three cases nothing was found. The remaining rabbit and dog which were inoculated have up to the present writing shown no symptoms of rabies, but these were inoculated subcutaneously, while the others were inoculated directly under the dura mater, and all died within twenty-one days. As the symptoms were almost exactly the same in all of the rabbits, and typical of rabies, we feel that we have clinched our diagnosis.

